# Multicopy targets for *Plasmodium vivax* and *Plasmodium falciparum* detection by colorimetric LAMP

**DOI:** 10.1186/s12936-021-03753-8

**Published:** 2021-05-19

**Authors:** Oscar Nolasco, Jhoel Montoya, Ana L. Rosales Rosas, Scarlett Barrientos, Anna Rosanas-Urgell, Dionicia Gamboa

**Affiliations:** 1 Instituto de Medicina Tropical “Alexander von Humboldt” Universidad Peruana Cayetano Heredia, Lima, Peru; 2 Unidad de Posgrado de la Facultad de Ciencias Biológicas Universidad Nacional Mayor de San Marcos, Lima, Peru; 3 Laboratorios de Investigación Y Desarrollo, Facultad de Ciencias Y Filosofía, Universidad Peruana Cayetano Heredia, Lima, Peru; 4 Department of Biomedical Sciences, Institute of Tropical Medicine, Antwerp, Belgium; 5 Departamento de Ciencias Celulares Y Moleculares, Facultad de Ciencias Y Filosofía, Universidad Peruana Cayetano Heredia, Lima, Peru

**Keywords:** Malaria, Molecular diagnosis, Colorimetric LAMP, *Pfr364*, *Pvr47*, *Cox1*, *PfEMP1*

## Abstract

**Background:**

Loop-mediated isothermal amplification (LAMP) for malaria diagnosis at the point of care (POC) depends on the detection capacity of synthesized nucleic acids and the specificity of the amplification target. To improve malaria diagnosis, new colorimetric LAMP tests were developed using multicopy targets for *Plasmodium vivax* and *Plasmodium falciparum* detection.

**Methods:**

The cytochrome oxidase I (*COX1*) mitochondrial gene and the non-coding sequence *Pvr47* for *P. vivax*, and the sub-telomeric sequence of erythrocyte membrane protein 1 (*EMP1*) and the non-coding sequence *Pfr364* for *P. falciparum* were targeted to design new LAMP primers. The limit of detection (LOD) of each colorimetric LAMP was established and assessed with DNA extracted by mini spin column kit and the Boil & Spin method from 28 microscopy infections, 101 malaria submicroscopic infections detected by real-time PCR only, and 183 negatives infections by both microscopy and PCR.

**Results:**

The LODs for the colorimetric LAMPs were estimated between 2.4 to 3.7 parasites/µL of whole blood. For *P. vivax* detection, the colorimetric LAMP using the COX1 target showed a better performance than the *Pvr47* target, whereas the *Pfr364* target was the most specific for *P. falciparum* detection. All microscopic infections of *P. vivax* were detected by PvCOX1-LAMP using the mini spin column kit DNA extraction method and 81% (17/21) were detected using Boil & Spin sample preparation. Moreover, all microscopic infections of *P. falciparum* were detected by Pfr364-LAMP using both sample preparation methods. In total, PvCOX1-LAMP and Pfr364-LAMP detected 80.2% (81 samples) of the submicroscopic infections using the DNA extraction method by mini spin column kit, while 36.6% (37 samples) were detected using the Boil & Spin sample preparation method.

**Conclusion:**

The colorimetric LAMPs with multicopy targets using the *COX1* target for *P. vivax* and the *Pfr364* for *P. falciparum* have a high potential to improve POC malaria diagnosis detecting a greater number of submicroscopic *Plasmodium* infections.

**Supplementary Information:**

The online version contains supplementary material available at 10.1186/s12936-021-03753-8.

## Background

Although significant progress has been achieved in malaria control and elimination over the last few decades, malaria remains a major public health problem, with 229 million cases worldwide in 2019 [1]. In Peru, 15,721 malaria cases were reported in 2020 [2], showing marked active transmission in rural and remote areas with poor access to medical care, despite the effort of the Peruvian malaria control programme (i.e., Plan Malaria Cero, PMC) to provide field-diagnosis based on microscopy detection and treatment to a large part of the affected population.

According to surveillance reports based on passive case detection and microscopy diagnosis, *Plasmodium vivax* causes four times more malaria infections than *Plasmodium falciparum* in the Peruvian Amazon region. However, such reports are based on passive case detection and microscopy assessment, which miss a high proportion of asymptomatic and submicroscopic infections that are only detected by molecular diagnosis [3, 4]. Infections that escape current malaria surveillance do not receive treatment and thus contribute to the maintenance of malaria transmission in communities [3].

Species-specific diagnosis is critical for adequate treatment in areas where both of the aforementioned species co-exist. In Peru, *P. falciparum* infections are treated using artemisinin-based combination therapy (ACT) with mefloquine and primaquine as partner drug, whereas *P. vivax* treatment is based on chloroquine to clear blood stages and primaquine to eliminate dormant liver stages (i.e., hypnozoites) that cause relapse [5].

Currently, the gold standard of malaria diagnosis in the field is still based on thin and thick blood smear and microscopy examination. In rural and remote areas, the major challenges that might lead to misdiagnoses are the lack of good diagnostic facilities, untrained microscopists, and the predominance of infections with parasitemia levels lower than 100 parasites/µL [6, 7]. In contexts where reliable microscopy is not available, diagnosis is done using rapid diagnostics tests (RDTs) based on monoclonal or polyclonal antibodies that detect malaria-specific antigens with a sensitivity comparable to microscopy [8]. However, several of these cannot detect low-density infections of *P. vivax* [9] or *HRP2*-negative *P falciparum* infections, which are reported in large proportions in Peru [10, 11].

PCR-based molecular diagnosis has shown better performance in detecting submicroscopic and asymptomatic infections in several studies carried out in Peru [12–15]. The majority of such studies have used PCR based on *18S rRNA* detection, which exists as four to eight copies in the *P. vivax* genome and five to eight copies in *P. falciparum* [16].

Despite the greater sensitivity of PCR-based techniques, their use at point of care (POC) is still challenging due to the dependence on expensive laboratory equipment and health workers’ technical capacity. On the other hand, molecular techniques based on isothermal amplification, such as loop-mediated isothermal amplification (LAMP), have the potential for the field diagnosis of malaria. LAMP allows for DNA amplification under isothermal conditions [17], and the results of the synthesis product can be detected visually by turbidity, change in colour reaction, or fluorescence [18, 19].

LAMP assays have been developed and evaluated to detect different *Plasmodium* species that cause human disease, and many of them with a limit of detection between 1 to 5 parasites/µL depending on the genomic target [20–30]. The majority of LAMPs use 18S rRNA and mitochondrial targets to detect pan genus *Plasmodium* or *P. falciparum* species. Most LAMP studies have been carried out in the Asian regions, with very few in South America [31, 32].

There are two commercial LAMP platforms that include a simplified system of sample preparation from blood and additional readout equipment, which improves its use as a point of care: the Loopamp MALARIA kits (Eiken Chemical Co. Ltd., Tokyo, Japan) for pan genus *Plasmodium*, *P. falciparum,* and *P. vivax* species detection [22]; and the Illumigene/Alethia Malaria LAMP (Meridian Bioscience, Inc., Cincinnati, OH, USA) [26] for pan genus *Plasmodium* detection. However, these kits are not routinely used due to their high cost and other operational challenges, such as lengthy sample preparation protocol.

In Peru, the commercial Loopamp™ MALARIA Pf detection and the Loopamp™ MALARIA Pv detection were evaluated in two different studies and showed a high operational capacity [33, 34]. Both studies were carried out without the reading equipment and showed crossed reactions in the species-specific identification. The cross-reaction of Loopamp™ MALARIA Pf detection kit showed a positive reaction in 3.9% (8/205) of *P. vivax* samples detected by PCR [33], and likewise, the Loopamp™ MALARIA Pv detection kit showed a positive reaction in 7.22% (13/180) of the *P. falciparum* samples detected by PCR [34].

A recent study in Peru evaluated a malachite green colorimetric LAMP (MG-LAMP) versus a fluorescence LAMP (RealAmp) using samples from symptomatic patients. Both LAMPs had problems detecting low parasitemia and mixed infections, specifically detecting *P. vivax* infection [32]. Hence, there is a need to assess alternatives targets in the LAMP assays. The best approach may be to use multicopy targets with genes with high copy number to improve sensitivity and detect low-density and mixed parasite infections [35, 36]. Recently, the *P. vivax Pvr47* gene and *P. falciparum Pfr364* gene (with 14 and 41 gene copies respectively) [37], were used in a real-time PCR assay and showed promise for identifying low-density *P. vivax* and *P. falciparum* infections and mixed infections, despite a variable ratio of species [38].

Erythrocyte membrane protein 1 (*EMP1*), present as several copies in the *P. falciparum* genome, has also been used in PCR and exhibits seven-fold higher sensitivity than the 18S ribosomal target [39]. Another very useful PCR target is the mitochondrial cytochrome oxidase 1 (*COX1*) [14]. In this study, we evaluated multicopy targets in *P. vivax* (*Pvr47* and *COX1*) and *P. falciparum* (*Pfr364* and *EMP1*) to improve diagnosis at POC. The targets were assessed by Colorimetric LAMP format using neutral red, pH-sensitive dye for the enhanced visual detection of the amplification product based on a rapid, distinct, and robust colour change from yellow to fuchsia due to the pH drop by the DNA target amplification. The performance of the method was evaluated using two different sample preparation protocols: the mini spin column kit (E.Z.N.A. ®Blood DNA Mini Kit) and the Boil & Spin method.

## Methods

### Primer LAMP design

First, we explored conserved regions of *PvCOX1*, *Pvr47*, *Pfr364*, and *PfEMP1* by aligning sequences available from PlasmoDB (plasmoDB.org) and the NIH genetic sequence database GenBank (https://www.ncbi.nlm.nih.gov/genbank/). *Pvr47* and *Pfr364* sequences were obtained from Demas et al. [37], composed of 14 *Pvr47* copies (*P. vivax* Sal-I strain) and 41 copies of *Pfr364* (subfamilies 1 and 2 of *P. falciparum* 3D7 strain). For the *COX1* and *EMP1* genes, we used the complete sequences reported in GenBank.

The LAMP primers were designed using the free software Primer Explorer V5 (http://primerexplorer.jp/lampv5e/index.html) and were located in a conserved region of each target. Only the primers for the *COX1* target were designed by checking manually the main point of primer design described in EIKEN GENOME SITE (http://loopamp.eiken.co.jp/e/lamp/primer.html). Primer specificity was tested using the NCBI Basic Local Alignment Tool (BLAST) tool https://www.ncbi.nlm.nih.gov/tools/primer-blast/) and potential secondary structures were analysed with OlygoAnalizer 3.1 (https://www.idtdna.com/calc/analyzer). Finally, four pairs of primers for each target were selected for LAMP assays (Table 1).

**Table 1:** Sequences of primers designed for *P. vivax* and *P. falciparum*

Specie	LAMP	Target		Primers Sequence (5ʹ→3 ʹ )
*P. vivax*	PvCOX1-LAMP	*COX1*	F3	GAATAATTGCACAAGAAAATGTTAAC
			B3	GCAACAGGAGATAAAGACATAAGTGA
			FIP	CAAGTTCTGGAGAACCACATAAAATTG– CCAGGATTATTTGGAGGATTCG
			BIP	GTCATTTTATCTACAGCAGCAGAATTT- TGGTGGATATAAAGTCCATCCAGT
	Pvr47-LAMP	*Pvr47*	F3	AACACCTCCCACCAATCA
			B3	GTGAATTATCGAAGGCATAA
			FIP	TACGCGGAAAATCAGAACAATTCAT- GTGCCAATTTTTTTTTGCGG
			BIP	ATCTTTCGCTTATCCATTCATCGA-TAGTGACAAAACATAAACACAGC
*P. falciparum*	PfEMP1-LAMP	*EMP1*	F3	CCGACAAAACTTTCACCCAA
			B3	CTGTTGTGTTGTTACCACTAGG
			FIP	GGTGTAACCACTATCAGTTCCACTA- AGTGGTAAATACAGAGGCAAAC
			BIP	TCCGAAAGTGAGTATGAAGAATTGG- ATGTTTTATATTTAGGACTACCTGG
	Pfr364-LAMP	*Pfr364*	F3	CACTAGGTACGCCAACAT
			B3	ACCCACAATTTTGATTGAGATG
			FIP	GTAGACACCATATGGTACCACGTA- GGATGTGTCTATCATATAGTCCG
			BIP	TTGTACCCAATTTTCCCCTAGCA- GTGTGTGCAACATCATAATCATCA

### LAMP isothermal conditions

The colorimetric LAMP assays were optimized to provide a clear visual detection based on the drop in pH caused by DNA target synthesis. When using the neutral red dye, the pH drop produces a change in the colour solution from yellow to fuchsia. For this, the reaction mixtures were prepared at a final volume of 25 μL, containing 0.5 × Bst DNA polymerase reaction buffer (10 mM Tris–HCl, 5 mM (NH_4_)_2_SO_4_, 25 mM KCl, 1 mM MgSO_4_, 0.05% Tween 20, pH 8.8 New England Biolabs Inc., MA, USA), 0.2 μM each external primer F3 and B3, 2 μM each inner primer FIP and BIP, 12U Bstv2.0 DNA polymerase (New England Biolabs Inc., MA, USA), 1.4 mM of each dNTPs, 0.003% of neutral red dye and 5 μL of a test sample. The reaction mixture for the Pvr47-LAMP was adjusted to 6 mM MgSO_4_, the PvCOX1-LAMP was adjusted to 6 mM MgSO_4_ and 0.4 M betaine, the Pfr364-LAMP was adjusted to 8 mM MgSO_4_ and the PfEMP1-LAMP was adjusted to 8 mM MgSO_4_ and 0.4 M betaine.

A positive reaction was assessed by visual inspection and considered as such only if the reaction turned from yellow to fuchsia after 65 min of incubation at 60 °C. Incubation steps were performed on a Bio-Rad T100 TM Thermal Cycler. (Bio-Rad Laboratories, CA, USA).

### Plasmid construction

To analyse the LOD of each colorimetric LAMPs in target copies, plasmids for each target were assembled through PCR amplification using the external primer F3/B3. The amplicons were cloned using a TOPO TA cloning kit (Life Technologies). Recombinant plasmid DNA was purified using a Qiagen plasmid mini kit, and the concentration of the extracted plasmid was determined using a Qubit dsDNA BR Assay Kit (ThermoFisher Scientific).

### Limit of detection

The LOD was assessed in two formats: (1) LOD in terms of number of copies per µL, for which serial dilutions of each recombinant plasmid with the specific target segment were used; and (2) LOD in terms of number of parasites per µL, for which serial dilutions of a cultured sample of *P. falciparum* 3D7 and pooled samples of whole blood from patients infected with *P. vivax* (quantified previously by microscopy and qPCR) were used. Each dilution series was tested 15 times by each colorimetric LAMP. The LOD was calculated using Probit regression whit the MedCalc Statistical Software version 19.2.6 (MedCalc Software bv, Ostend, Belgium; https://www.medcalc.org; 2020).

### Reference samples

The reference samples from the *P. falciparum* 3D7 culture and a pool of blood samples infected with *P. vivax* (microscopy and PCR-confirmed) were supplied by the malaria laboratory at the Universidad Peruana Cayetano Heredia (UPCH). DNA controls were supplied by the Centers for Disease Control and Prevention (CDC). The quality control distribution 4260 of WHO and UK National External Quality Assessment Service (UK NEQAS) Parasitology were used for validation of genus and species. The 4260 material contained five samples: specimen 4307 (*Plasmodium ovale* 200,000 parasites/mL), specimen 4308 (*Plasmodium vivax* 20,000 parasites/mL), specimen 4309 (*P. falciparum* 20,000 parasites/mL), specimen 4310 (No *Plasmodium* nucleic acid detected), and specimen 4311 (*Plasmodium knowlesi* 200,000 parasites/mL).

### Clinical samples

A total of 312 blood samples were selected from the sample bank of the Malaria Laboratory at UPCH. The samples were collected from 10 communities (Quistococha, Santo Tomas, Rumococha, Gamitanacocha, Libertad, 1 de Enero, Salvador, Lago Yuracyacu, Puerto Alegre, and Urcomiraño) from Loreto (Peru) during an active sample collection in 2018. All samples were collected after an informed consent form was signed according to the ethical clearance from the Ethics Review Board of the UPCH, Lima, Peru (SIDISI code 101645).

**Fig. 1:**
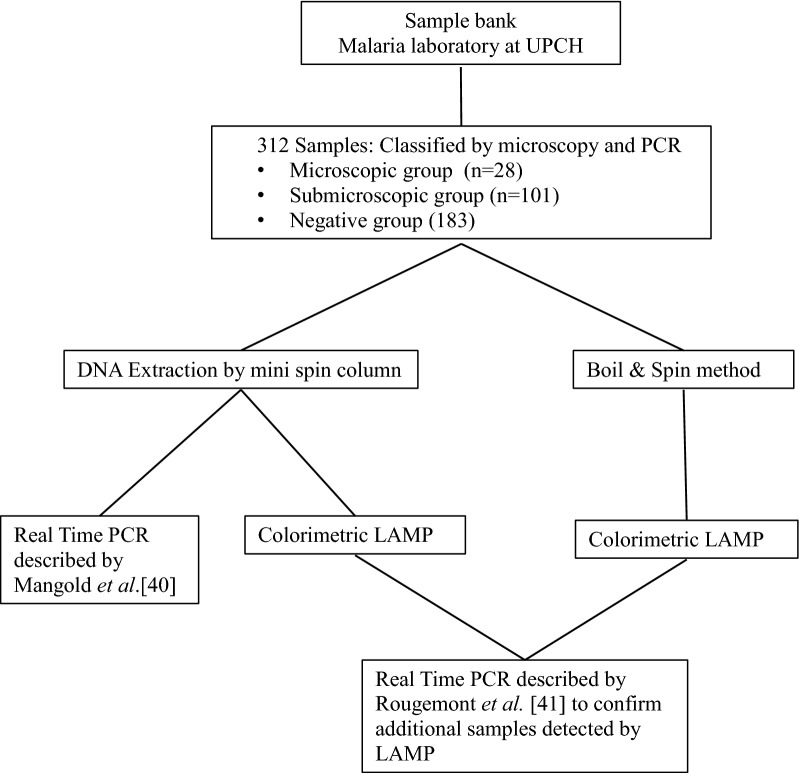
Flowchart of the experimental procedure

These samples were categorized into three groups. The first group included 28 samples with infections detected by both microscopy and real-time PCR with 21 *P. vivax* infections and 7 *P. falciparum* infections. All samples showed asexual parasitaemia with more than 100 parasites/µL as assessed by microscopy, with the exception of three *P. vivax* infections that had < 100 parasites/µL. The second group included 101 submicroscopic infections (83 *P. vivax* and 18 *P. falciparum* infections) detected only by real-time PCR as described by Mangold et al*.* [40]. The third group included 183 negative samples as determined by both microscopy and real-time PCR; this group of samples was included to assess interference of human DNA with the LAMP primers. Figure 1 shows the flowchart of the experimental procedure and details of the diagnosis of clinical samples are available in Additional file 1.

### Microscopy data

All the selected samples have a result of the thick drop and thin film for malaria diagnosis. Parasite densities were quantified by counting asexual parasites and gametocytes separately with the number of thick drop leukocytes based on an estimated mean count of 8000 leukocytes per microliter of blood. The counting criteria were applied according to the Peruvian Minister of Health [5] and were carried out with the following formula:

$$ \frac{{{\text{N}}^\circ {\text{ Parasites}}}}{{{\text{N}}^\circ {\text{ Leukocytes}}}} \times 8000 = {\text{Parasites}}/\upmu {\text{L}} $$

### DNA extraction by mini spin column

The DNA was extracted using the EZNA® Blood DNA (Omega Bio-tek, Norcross, GA, USA), following the manufacture’s protocol. In brief, 40 µL blood was transferred to a sterile microcentrifuge tube adding 250 µL 2X TEN buffer pH 8.0 (0.2 M NaCl, 0.02 M Tris–HCl pH 8.0, 0.002 M EDTA pH 8.0), 180 µL distilled water, 50 µL of 10% SDS; and 25 µL OB protease solution. The mix was incubated at 65 °C for 60 min and centrifuged at 13,000*g* for 5 min; the supernatant was transfer to a new tube, adding 250 µL of BL buffer and 260 µL of 100% ethanol. Then, 500 µL of this homogenate was transferred to a mini HiBind® DNA column inserted into a 2 mL collecting tube and centrifuged at 10,000*g* for 1 min. The filtrate was discarded, and the mini column was inserted into a new 2 mL collection tube, 500 µL of HBC buffer was added, then centrifuged at 10,000*g* for 1 min and the filtrate was discarded. Then, 700 µL of wash buffer was added to the column and centrifuged at 10,000*g* for 1 min; this process was repeated once more. The empty HiBind mini DNA column was centrifuged at maximum speed for 2 min to dry the column matrix. The DNA from the column was eluted in 60 µL elution buffer preheated to 65 °C. Eluted DNA was stored at − 20 °C. Hereafter, the DNA extracted using this method is referred to as MSC DNA.

### Sample preparation using the boiling & spin extraction method

The Boil & Spin method was performed using 40 µL 2X lysis buffer (8 mM Tris, 80 mM NaCl, 0.08% SDS, pH 8.5) and 40 µL the blood sample. The mixture was homogenized and incubated in a water bath at 95 °C for 7 min. Next, the homogenate was centrifuged at 13,000*g* for 7 min. Finally, the supernatant was recovered and used for the LAMP analysis. Hereafter, the DNA extracted using this method is referred to as BS DNA.

### Species-specific detection by real-time PCR

The 18S rRNA gene was chosen for species-specific detection using the qPCR performed by Mangold et al*.* [40]. The reaction mixtures were prepared at a final volume of 25 µL with a final concentration of 1X master mix of PerfeCTa SYBR® Green Fastmix, and 0.3 μM each primer PL1473F18 5′-TAACGAACGAGATCTTAA-3′ and PL1679R18 5′-GTTCCTCTAAGAAGCTTT-3′ [40].

The PCR conditions consisted of an initial denaturation step at 95 °C for 2 min, followed by 45 cycles of 20 s at 95 °C, 20 s at 50 °C, and 20 s at 68 °C, with fluorescence acquisition at the end of each extension step. Amplification was immediately followed by a melting curve program consisting of 2 min at 68 °C, and a stepwise temperature increase of 0.5 °C/s until 90 °C, with fluorescence acquisition at each temperature transition. All of the samples with a quantification cycle (Cq) of less than 36.2 were considered positive according to the LOD determined using a serial dilution of the 1st WHO International Standard for *Plasmodium* falciparum DNA Nucleic Acid Amplification Techniques (NIBSC code 04/176) available at the malaria laboratory, and the same LOD for *P. vivax* was assumed, considering that it is the same amplification target. The melting curve analysis was used to determine the species-specific: a melting peak of 73.5 °C ± 0.5 was considered to define *P. falciparum* infection and a peak of 77 °C ± 0.5 was considered to determine *P. vivax* infection.

### Statistical analyses

Correlation between the diagnotics test were evaluated using the Cohen kappa test. Analyses of sensitivity and specificity were performed using the free software package reportROC in RStudio.

## Results

### LAMP analytical sensitivity and specificity

Using a serial dilution of plasmid for each target, Pfr364-LAMP showed an LOD of 27.3 copies of plasmid/µL and PfEMP1-LAMP showed an LOD of 28.9 copies of plasmid/µL. For *P. vivax* detection, PvCOX1-LAMP showed an LOD of 23.9 copies of plasmid/µL and the Pvr47-LAMP showed an LOD of 15.6 copies of plasmid/µL (Table 2). In addition, sensitivity analyses were performed using serial dilutions from a 3D7 cultured blood sample for *P. falciparum* to define LOD based on parasites/µL*.* The results showed an LOD of 3.7 parasites/µL for Pfr364-LAMP and of 3.3 parasites/µL for PfEMP1-LAMP. For *P. vivax*, using a pooled samples of blood from infected patients (quantified as parasite/µL by PCR), the PvCOX1-LAMP showed an LOD of 2.4 parasites/µL and the Pvr47-LAMP showed an LOD of 3 parasites/µL (Table 2).

**Table 2:** Limit of detection

Using blood samples		Using recombinant plasmid by each target	
PvCOX1-LAMP LOD 2.4 parasites/μL 95%CI 1.8–4.8		PvCOX1-LAMP LOD 23.9 copies/μL 95%CI 16.1–65.8	
Parasites/μL^a^:	Positive/n° replicates	Plasmid (Copies/μL):	Positive/n° replicates
214.1	15/15	1684.0	15/15
21.4	15/15	168.4	15/15
2.1	13/15	16.8	13/15
1.1	9/15	8.5	7/15
0.5	0/15	3.4	1/15

Pvr364-LAMP LOD 3.7 parasites/μL 95%CI 2.8–7.5		Pvr364-LAMP LOD 27.3 copies/μL 95%CI 19.1–133.6	
3D7 culture (Parasites/μL):	Positive/n° replicates	Plasmid (Copies/μL):	Positive/n° replicates
314.2	15/15	1865.5	15/15
31.4	15/15	186.6	15/15
3.1	13/15	18.7	12/15
1.6	6/15	9.3	4/15
0.8	0/15	1.9	0/15

Pvr47-LAMP LOD 3 parasites/μL 95%CI: 2.1–6.8		Pvr47-LAMP LOD 15.6 copies/μL 95%CI: 11.9–30.5	
Parasites/μL^a^:	Positive/n° replicates	Plasmid (Copies/μL):	Positive/n° replicates
214.1	15/15	1631.6	15/15
21.4	15/15	163.2	15/15
2.1	12/15	16.3	14/15
1.1	5/15	8.2	9/15
0.5	0/15	3.3	0/15

PfEMP1-LAMP LOD 3.3 parasites/μL 95%CI: 2.4–6.7		Pvr47-LAMP LOD 28.9 copies/μL 95%CI: 21.2–86.7	
3D7 culture (Parasites/μL):	Positive/n° replicates	Plasmid (Copies/μL):	Positive/n° replicates
314.2	15/15	2076.0	15/15
31.4	15/15	207.6	15/15
3.1	14/15	20.8	12/15
1.6	8/15	10.4	3/15
0.8	1/15	2.1	0/15

^a^Pool of blood samples from infected patients quantified in parasite/µL by real-time PCR

**Fig. 2:**
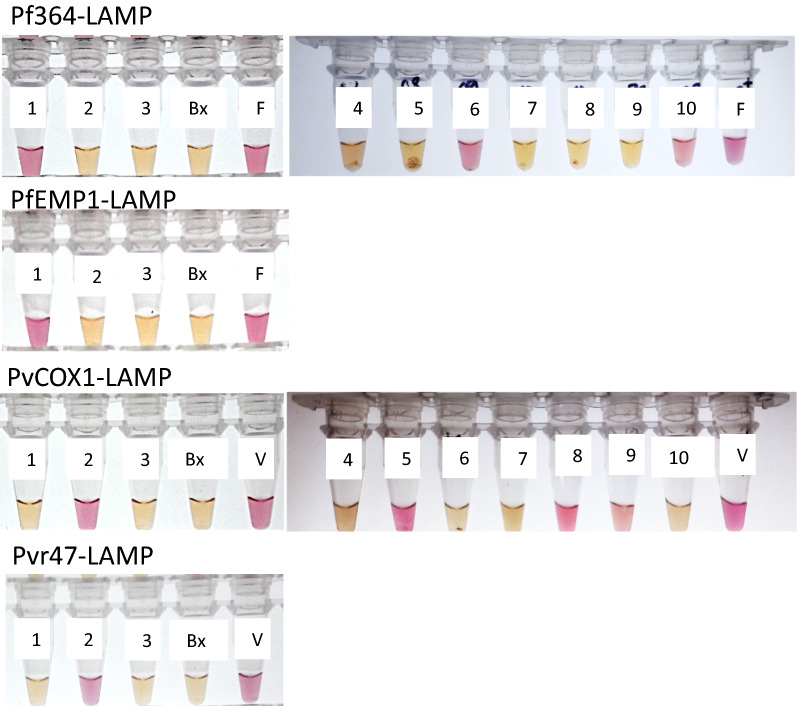
Specificity of the different colorimetric LAMPs. (1) *P. falciparum* 3D7; (2) *P. vivax* Sal-I; (3) *P. malariae*; (4) *P*. *ovale* 200,000 parasites/mL, (5) *P*. *vivax* 20,000 parasites/mL, (6) *P*. *falciparum* 20,000 parasites/mL, (7) No *Plasmodium* nucleic acids detected, (8) *P*. *knowlesi* 200,000 parasites/mL, (9) *P*. *vivax* 10,000 parasites/mL, (10) *P*. *falciparum* 10,000 parasites/mL, F) *P*. *falciparum* positive control, V) *P*. *vivax* positive control, BX) blank control

Overall, colorimetric LAMPs with *PfEMP1* and *Pfr364* targets showed high specificity for detecting *P. falciparum* 3D7 and the corresponding reference samples. These results are shown in Fig. 2. The colorimetric LAMPs designed for *P. vivax* with the *COX1* and *Pvr47* targets showed a positive reaction with *P. vivax* strain Sal-I, but it also showed a positive reaction with *P. knowlesi,* likely due to the high genomic similarity.

### *Plasmodium vivax* detection using the *COX1* and *Pvr47* multicopy targets

The PvCOX1-LAMP displayed the best sensitivity for *Plasmodium vivax* detection. Using MSC DNA, the PvCOX1-LAMP detected 100% (21/21) of microscopic samples and 86.7% (72/83) of submicroscopic samples, whereas the Pvr47-LAMP detected only 71.4% (15/21) and 36.1% (30/83) of these samples, respectively (Table 3). Likewise, using BS DNA, the PvCOX1-LAMP detected 81% (17/21) of microscopic samples and 36.1% (30/83) of submicroscopic samples. The Pvr47-LAMP only detected 52.4% (11/21) and 22.9% (19/83) of these samples, respectively. In the microscopic infection group, only four BS samples produce negative results in PvCOX1-LAMP; two of these samples had parasite densities of 324 and 514 parasites/µL, while the other two showed the lowest parasite density detected by microscopy with 32 parasites/µL.

**Table 3:** Sensitivity and specificity of *Pvr47* and *COX1* targets to detect *P. vivax*

	Mini spin column				Boil & Spin			
	Pvr47-LAMP		PvCOX1-LAMP		Pvr47-LAMP		PvCOX1-LAMP	
*P. vivax* infection detected by microscopy & PCR	71.4%	95 CI 47.7–87.8%	100.00%	95 CI 80.8–100%	52.4%	95 CI 30.3–73.6%	81.0%	95 CI 57.42–93.71%
n = 21	n = 15		n = 21		n = 11		n = 17	
*P. vivax* submicroscopic infection detected only by PCR	36.1%	95 CI 26.1–47.5%	86.8%	95 CI 77.1–92.9%	22.9%	95 CI 14.7–33.7%	36.1%	95 CI 26.1%–47.5%
n = 83	n = 30		n = 72		n = 19		n = 30	
Non *P. vivax* infection detected by microscopy & PCR	95.7%	95 CI 91.7–97.9%	97.6%	95 CI 94.2–99.1%	97.1%	95 CI 93.5–98.8%	100%	95 CI 97.7–100%
Negatives = 208	n = 199		n = 203		n = 202		n = 208	
Sensitivity^a^	43.3%	95 CI: 33.7%-52.8%	89%	95 CI: 83.5%- 95.3%	29%	95 CI: 20.1%-37.6%	45%	95 CI: 35.6%- 54.8%
Specificity^a^	95.7%	95CI 92.9–98.4%	98%	95 CI 95.5–99.7%	97%	95CI 94.8–99.4%	100%	100%
Agreement Cohen’s Kappa^a^	0.443	0.338–0.547	0.883	0.827–0.939	0.310	0.208–0.412	0.524	0.425–0.622

^a^Reference method: real-time PCR described by Mangold et al. [40]

**Fig. 3:**
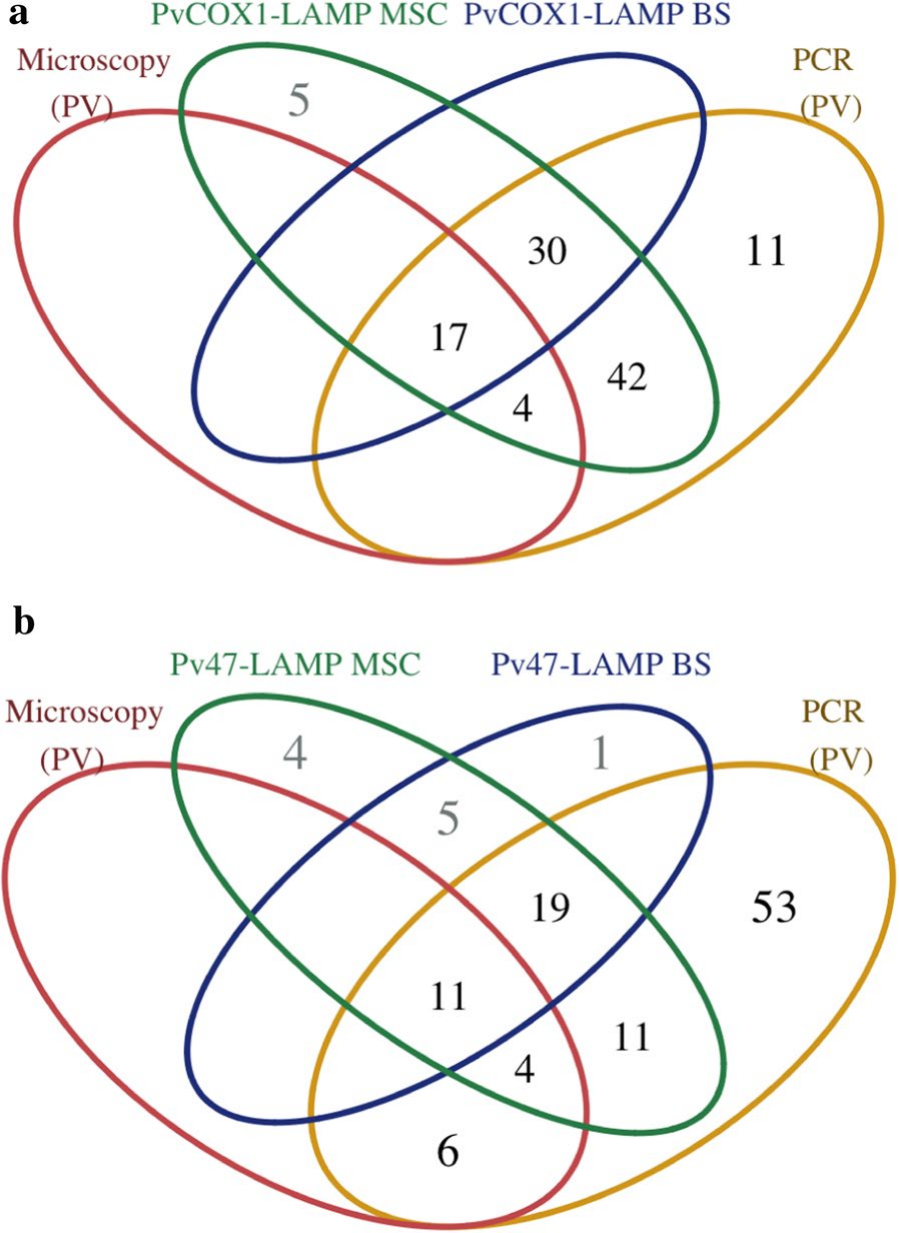
Venn diagram of the *P. vivax* detection. **a** PvCOX1-LAMP comparison with microscopy and PCR. **b** Pvr47-LAMP comparison with microscopy and PCR. *MSC* Mini-Spin Column, *BS* Boil & Spin

Overall, 11 of 83 submicroscopic *P. vivax* infections (13.3%) were negative in PvCOX1-LAMP using sample from both preparation methods (see Additional file 1). Of these samples, one quantified by PCR with 6 parasites/µL was above the 95% confidence interval of the LOD (1.8–4.8 parasites/µL), another six samples had parasitaemia values within the interval and four had parasitaemia below this range. Finally, 30 submicroscopic infections were also detected by PvCOX1-LAMP using both types of extracted DNA, whereas 42 were detected only using MSC DNA (Fig. 3a).

Five additional positive samples to *P. vivax* by PvCOX1-LAMP were found in the third group of negative samples, by using MSC DNA (Fig. 3a). Upon further examination, these samples were considered to have had lower parasitaemia levels to *P. vivax*, with the Taqman PCR, described by Rougemont et al*.* [41]. In addition, in Pv47-LAMP, nine samples were positive for *P. vivax* using MSC DNA, five of them were also positive using BS DNA, and one additional sample was positive using BS DNA (Fig. 3b). However, negative results were always obtained for these 10 samples using different real-time PCR methods.

### *Plasmodium falciparum* detection using the *EMP1* and *Pfr364* multicopy targets

The sensitivity and specificity of the *Pfr364* and the *PfEMP1* targets were similar using all three groups of samples and the PCR as a reference method. Both LAMPs showed a moderate agreement, with Cohen kappa indexes between 0.74–0.82 when using MSC DNA and 0.64–0.57 when using BS DNA (Table 4).

**Table 4:** Sensitivity and specificity of Pfr364 and EMP1 targets to detect *Plasmodium falciparum*

	Mini spin column				Boil & Spin			
	Pfr364-LAMP		PfEMP1-LAMP		Pfr364-LAMP		PfEMP1-LAMP	
*P. falciparum* infection detected by microscopy & PCR	100.0%	95 CI 56.1–100%	100.0%	95 CI 56.1–100%	100.0%	95 CI 56.1–100%	100.0%	95 CI 56.1–100%
n = 7	n = 7		n = 7		n = 7		n = 7	
*P. falciparum* submicroscopic infection detected only by PCR	50.0%	95 CI 29.0–71%	72.2%	95 CI 46.4–89.3%	33.3%	95 CI 14.4–58.9%	22.2%	95 CI 7.4–48.1%
n = 18	n = 9		n = 13		n = 6		n = 4	
Non *P. falciparum* infection detected by microscopy & PCR	99.7%	95 CI 97.8–100%	99.0%	95 CI 96.8–99.7%	99.7%	95 CI 97.8–100%	99.7%	95 CI 97.8–100%
n = 287	n = 286		n = 284		n = 286		n = 286	
Sensitivity^a^	64.0%	95 CI 45.2–82.8%	80.0%	95 CI 64.3–95.7%	52.0%	95 CI 32.4–71.6%	44.0%	95 CI 24.5–63.5%
Specificity^a^	99.7%	95CI 99–100%	99.0%	95 CI 97.8–100%	99.7%	95CI99–100%	99.7%	95CI 99–100%
Agreement Cohen’s Kappa^a^	0.745	0.595–0.896	0.820	0.698–0.941	0.646	0.470–0.823	0.572	0.381–0.764

^a^Reference method: real-time PCR described by Mangold et al. [40]

**Fig. 4:**
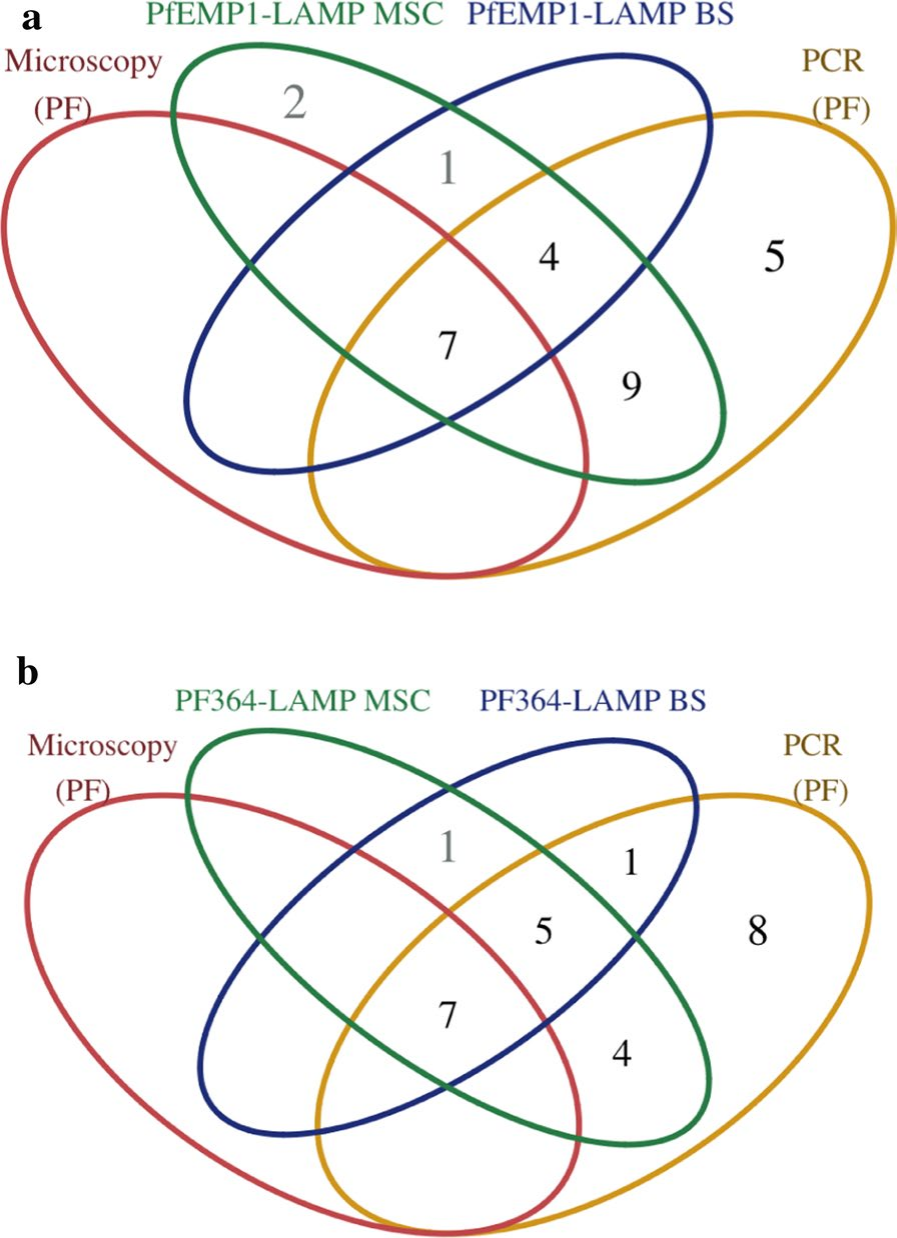
Venn diagram of the *P. falciparum* detection by diagnostic method. **a** PfEMP1-LAMP comparison with microscopy and PCR. **b** Pfr364-LAMP comparison with microscopy and PCR. *MSC* Mini-Spin Column, *BS* Boil & Spin

Both, Pfr364-LAMP and the PfEMP1-LAMP detected all infections positive that were identified by microscopy (n = 7) using DNA from both sample preparation methods. In the submicroscopic group, two of nine *P. falciparum* infections with parasitemia above the 95% confidence interval of Pfr364-LAMP LOD (2.8–7.5 parasites/µL) showed a negative result when tested by Pfr364-LAMP using MSC DNA. One submicroscopic infection with parasitemia above the 95% confidence interval of PfEMP1-LAMP LOD (2.4–6.7 parasites/µL) showed a negative result when tested by PfEMP-LAMP using MSC DNA. However, both assays exhibited difficulty detecting submicroscopic infections using BS DNA. Moreover, the Pfr364-LAMP detected 6 out of 18 PCR positive samples, whereas PfEMP1-LAMP detected only four samples (Fig. 4). One *P. vivax* infection detected by PCR with low parasitaemia of 40 parasites/µL was additionally detected as *P. falciparum* infection by the two LAMPs with both types of extracted DNA. This sample was also positive for *P. vivax* in PvCOX1-LAMP using MSC DNA, and later it was confirmed by Taqman PCR (as described by Rougemont et al. [41]) to be a mixed sample.

In PfEMP1-LAMP using MSC DNA, two additional *P. falciparum* were detected. One of these was a *P. vivax* submicroscopic infection, and the other one was a negative sample.

### *Plasmodium vivax* and *P. falciparum* detection

Considering the *P. vivax* and *P. falciparum* diagnosis scenario with two individual LAMP assays and the PCR as a reference method, PvCOX1-LAMP and Pfr364-LAMP together showed a sensitivity of 84.5% compared to the 87.6% sensitivity of both PvCOX1-LAMP and PfEMP1-LAMP together using MSC DNA; the values using BS DNA were 47.3% and 45.7%, respectively (Table 5).

**Table 5:** Sensitivity and specificity for *P. vivax* and *P. falciparum* detection

	Mini Spin Column				Boil & Spin			
	PvCOX1-LAMP & Pfr364-LAMP		PvCOX1-LAMP & PfEMP1-LAMP		PvCOX1-LAMP & Pfr364-LAMP		PvCOX1-LAMP & PfEMP1-LAMP	
*P. vivax* and *P. falciparum* infection detected by microscopy & PCR	100.0%	95 CI 85–100%	100.0%	95 CI 85–100%	85.7%	95 CI 66.4–95.3%	85.7%	95 CI 66.4–95.3%
n = 28	n = 28		n = 28		n = 24		n = 24	
*P. vivax* and *P. falciparum* submicroscopic infection detected only by PCR	80.2%	95 CI 70.8–87.2%	84.2%	95 CI 75.3–90.4%	36.6%	95 CI 27.4–46.86%	34.7%	95 CI 25.64–44.9%
n = 101	n = 81		n = 85		n = 37		n = 35	
Non *P. vivax* and *P. falciparum* infection detected by Microscopy & PCR	97.3%	95 CI 93.4–99%	96.7%	95 CI 92.7–98.7%	100.0%	95 CI 97.4–100%	100.0%	95 CI 97.4–100%
n = 183	n = 178		n = 177		n = 183		n = 183	
Sensitivity^a^	84.5%	95 CI 78.3–90.7%	87.6%	95 CI 81.9–93.3%	47.3%	95 CI 38.7–55.9%	45.7%	95 CI 37.1–54.3%
Specificity^a^	97.3%	95CI 94.9–99.6%	96.7%	95 CI 94.1–99.3%	100.0%	95CI 100–100%	100.0%	95CI 100–100%
Agreement Cohen’s Kappa^a^	0.832	0.769–0.895	0.853	0.794- 0.912	0.513	0.423–0.602	0.497	0.407–0.587

^a^Reference method: real-time PCR described by Mangold et al.[40]

PvCOX1-LAMP and Pfr364-LAMP combined system displayed slightly more specificity than PvCOX1-LAMP and PfEMP1-LAMP combination using MSC DNA, at 97.3% and 96.7% respectively (Table 5). This slight difference indicates that PfEMP1-LAMP may decrease the specificity of the system when the two individual LAMP assays are used for both *P. vivax* and *P. falciparum* detection.

## Discussion

In Peru, the highest incidence of malaria cases occurs in rural regions where diagnosis by microscopy is still used. However, these areas carry additional challenges, such as the lack of expert microscopy technicians in peripheral health centers and poor maintenance of microscope equipment, which jeopardizes the quality of the results.

In this study, new colorimetric LAMP assays, which were tested with microscopic and submicroscopic samples previously evaluated by real-time PCR. Of the different colorimetric formats available for the visualization of LAMP results, colour change using pH indicators, such as neutral red, is advantageous compared to dyes such as malachite green, which can vary in colour intensity and bias the visualization to yield unsatisfactory results [42].

In laboratory conditions, the colorimetric LAMPs showed an LOD between 2.4 and 3.7 parasites/µL. This detection level is comparable with other LAMP studies for malaria diagnosis, in which the sensitivity has varied according to the target employed. Several studies have reported LODs of 10–125 parasites/µL using genus-specific and species-specific tests with targets such as 18S rRNA target, alpha-tubulin, and r64 repeat sequences [43–45]. A lower LOD (between 1.4 and 5 parasites/µL) was reached using the species-specific test for *P. vivax* and *P. falciparum* with a multicopy mitochondrial target [20, 23, 46].

Four *P. vivax* microscopic infections with parasitaemia higher than the LOD determined for PvCOX1-LAMP were negative when analysed by BS DNA. These samples were four out of the six samples with the lowest parasitaemia in the microscopic infection group, and the negative result could have been affected by inhibitors that the sample preparation method could not remove. Likewise, one *P. vivax* submicroscopic infection with parasitaemia above the 95% confidence interval of the PvCOX1-LAMP LOD (1.8–4.8 parasites/µL) showed a negative result when tested using PvCOX1-LAMP paired with MSC DNA. Similarly, two of nine *P. falciparum* submicroscopic infections with parasitaemia above the 95% confidence interval of Pfr364-LAMP LOD (2.8–7.5 parasites/µL) showed a negative result when tested by Pfr364-LAMP paired with MSC DNA. These negative results significantly increased when BS DNA was used for this submicroscopic group. In fact, the false negatives with a parasitaemia level close to the LOD could be explained by the low DNA quality, which was lower using the Boil & Spin method due to enzyme inhibitors.

The designed LAMP assays have proven to be highly specific using samples of different types of *Plasmodium*. Only the *COX1* target showed reactivity for *P. vivax* and *P. knowlesi,* most likely because the two species are genetically related; however, this disadvantage only represents a problem in Asian countries where *P. knowlesi* is more predominant [47]. Despite that the *Pvr47* target demonstrating high specificity and sensitivity in detecting *P. vivax* by real-time PCR [37], the Pvr47-LAMP designed in this study showed lower *P. vivax* detection compared to the PvCOX1-LAMP, using either MSC or BS DNA. Thus, future evaluation is necessary to determine a possible cause, such as a mismatched primer in this multicopy target.

Comparing the sample preparation methods, the use of Boil & Spin method was simple, fast and did not require much experience, in contrast the commercial mini spin column kit required some experience in handling laboratory materials and special storage conditions. In addition, the reagents were less expensive using the formed method: $ 0.03 USD per extraction for Boil & Spin and $ 2.70 USD per extraction for the mini spin column kit. However, the performance of the formed decreased when the amplification target was present in low quantities and due to the presence of enzyme inhibitors that could not be eliminated.

Overall, the results with the malaria microscopic infections group indicate that LAMP assays whit MSC DNA can be used instead of the microscopy diagnosis, because all the microscopic samples tested gave a positive reaction with the respective LAMP assay (PvCOX1-LAMP for *P. vivax* and PfEMP1-LAMP or Pfr364-LAMP for *P. falciparum*). Similarly, assays using BS DNA also showed high performance, with only four such samples testing negative for *P. vivax*. Nonetheless, a future study with a greater number is needed to determine if the Boil & Spin method can be used when diagnostic by microscopy cannot be performed with the good requirements of quality.

The PvCOX1-LAMP detected five samples positive for *P. vivax* that were negative in the real-time PCR described by Mangold et al*.* [40]. Indeed, these were all confirmed to be *P. vivax* infections using the Taqman PCR described by Rougemont et al. [41]; they had very low parasite loads at 5 parasites/µL. This discrepancy was probably due to stochastic amplification occurring at low parasite densities.

As noted above, both Pfr364-LAMP and PfEMP1-LAMP detected all *P. falciparum* microscopic infections with both types of DNA extracted. However, they both showed the same difficulties in detecting submicroscopic infections below 10 parasites/µL (see Fig. 4 and Additional file 1). Unfortunately, the number of samples evaluated for *P. falciparum* was very small, which prevented from defining the best test for a suitable routine evaluation of this species. However, based on the results of species identification, the more specific assay for detecting *P. falciparum* was Pfr364-LAMP.

In the context of malaria diagnosis in the field, the PMC promotes the training of microscopists to achieve quality and prompt diagnosis to give specific treatment. However, the species-specific diagnosis of submicroscopic infections remains the main challenge. The LAMP techniques would greatly complement microscopy diagnostic by detecting an essential group of submicroscopic infections with high specificity, as demonstrated by the results of this study.

These newly developed colorimetric LAMP assays have significant advantages, such as the low cost and a straightforward interpretation of results based on the naked-eye visual inspection. These assays should be further evaluated in the field and at POC to determine field implementation logistics and healthcare worker training requirements. As mentioned above, the use of Boil & Spin or further simplified DNA methods would improve the malaria diagnosis at POC in settings with poor-quality microscopic diagnostic conditions. Moreover, an additional investment to use mini spin column kits would allow for a better diagnosis by detecting many submicroscopic infections.

## Conclusion

The simultaneous use of *COX1* and Pfr364 multicopy target in colorimetric LAMP for both *P. vivax* and *P. falciparum* detection improved diagnosis detecting 37(36.63%) and 81(80.20%) submicroscopic infections using a simple method of sample preparation (Boil & Spin) or a mini column spin kit, respectively.

## Supplementary Information

**Additional file 1.** Malaria Diagnosis data.

## Data Availability

The datasets generated during and/or analysed during the current study are available from the corresponding author on reasonable request.

## Abbreviations

BLAST: Basic Local Alignment Tool
COX1: Cytochrome oxidase I
DNA: Desoxyribonucleic acid
EDTA: Ethylene diamine tetra-acetic acid
EMP1: Erythrocyte membrane protein 1
HRP2: Histidine-rich protein 2
LAMP: Loop-mediated Isothermal amplification
LOD: Limit of detection
MG-LAMP: Malachite green colorimetric LAMP
NIH: National Institute of Health
NCBI: National Center for Biotechnology Information
PCR: Polymerase chain reaction
PMC: Plan Malaria Cero
POC: Point of care
RDTs: Rapid diagnostics tests
SDS: Sodium dodecyl sulfate
TEN buffer: Tris–EDTA Sodium chloride buffer
18S rRNA: Ribosomal sub-unit small 18S RNA
